# Transcriptome Characterization and Expression Analysis of Chemosensory Genes in *Chilo sacchariphagus* (Lepidoptera Crambidae), a Key Pest of Sugarcane

**DOI:** 10.3389/fphys.2021.636353

**Published:** 2021-03-05

**Authors:** Jianbai Liu, Huan Liu, Jiequn Yi, Yongkai Mao, Jihu Li, Donglei Sun, Yuxing An, Han Wu

**Affiliations:** ^1^Guangdong Engineering Research Center for Pesticide and Fertilizer, Institute of Bioengineering, Guangdong Academy of Sciences, Guangzhou, China; ^2^College of Horticulture and Plant Protection, Henan University of Science and Technology, Luoyang, China

**Keywords:** *Chilo sacchariphagus*, transcriptome, chemosensory genes, gene expression, phylogenetic analysis

## Abstract

Insect chemoreception involves many families of genes, including odourant/pheromone binding proteins (*OBP/PBPs*), chemosensory proteins (*CSPs*), odourant receptors (*ORs*), ionotropic receptors (*IRs*), and sensory neuron membrane proteins (*SNMPs*), which play irreplaceable roles in mediating insect behaviors such as host location, foraging, mating, oviposition, and avoidance of danger. However, little is known about the molecular mechanism of olfactory reception in *Chilo sacchariphagus*, which is a major pest of sugarcane. A set of 72 candidate chemosensory genes, including 31 *OBPs/PBPs*, 15 *CSPs*, 11 *ORs*, 13 *IRs*, and two *SNMPs*, were identified in four transcriptomes from different tissues and genders of *C. sacchariphagus*. Phylogenetic analysis was conducted on gene families and paralogs from other model insect species. Quantitative real-time PCR (qRT-PCR) showed that most of these chemosensory genes exhibited antennae-biased expression, but some had high expression in bodies. Most of the identified chemosensory genes were likely involved in chemoreception. This study provides a molecular foundation for the function of chemosensory proteins, and an opportunity for understanding how *C. sacchariphagus* behaviors are mediated via chemical cues. This research might facilitate the discovery of novel strategies for pest management in agricultural ecosystems.

## Introduction

Insects, the most diverse and successful group of animals on earth, have existed for more than 350 million years (Stork, 1993; Chen et al., 2018); they not only affect the natural environment but also influence human life and productivity in many ways. A sophisticated chemosensory system makes insect prominence among other animals for their survival and reproduction (Leal, 2013). Chemoreception plays a critical role in many insect behaviors, including behaviors to avoid harm from predators or the surrounding environment, behaviors to detect locations for oviposition or hosts, searching for food or mates, and interspecific communication (Stocker, 1994; Hildebrand, 1995; Grosse-Wilde et al., 2011; Zhang et al., 2015). The recognition of chemical signals depends on peripheral chemosensory systems (Vieira and Rozas, 2011; Zhang et al., 2016). External chemical ligands are recognized by binding and membrane receptor proteins located in the antennae, which have many kinds of sensilla, and then translated into electrical signals to the central nervous system (Robertson et al., 2003; Ramdya and Benton, 2010). Chemoreception in insects is mediated via many proteins, including odourant binding proteins (OBPs), pheromone binding proteins (PBPs), chemosensory proteins (CSPs), odourant receptors (ORs), ionotropic receptors (IRs), and sensory neuron membrane proteins (SNMPs) (Leal, 2013; Pelosi et al., 2014, 2018; Wicher, 2014; Butterwick et al., 2018; He et al., 2019b).

Insect OBPs, small water-soluble proteins with molecular masses of approximately 14 kDa that were first found in *Antheraea polyphemus* (Vogt and Riddiford, 1981), are present at high concentrations in the sensillum lymph (Vogt and Riddiford, 1981; Pelosi et al., 2006). OBPs act as a liaison between external chemicals and ORs (Leal, 2005), recognizing hydrophobic odourants and delivering them to olfactory receptors (ORs) on olfactory sensory neurone (OSN) membranes (Pelosi et al., 2006; Xu et al., 2009; Leal, 2013), which is the first and key step in the process of olfaction. CSPs, which were found to be soluble binding proteins (Gong et al., 2007), are abundant in the sensillum lymph (Vogt and Riddiford, 1981; Prestwich, 1996; Pophof, 2004; Grosse-Wilde et al., 2006; Lautenschlager et al., 2007; Leal, 2007; Laughlin et al., 2008; Kaissling, 2009) and also expressed in many organs and tissues, such as antennae, wings, legs, maxillary palps, and labial palps, with the function of affecting chemoreception (Jacquinjoly et al., 2001; Jin et al., 2005; González et al., 2009; Pelletier and Leal, 2011; Gu et al., 2013; Zhang et al., 2014). PBPs, a kind of special odor-binding protein that can dissolve and transport fat-soluble pheromones through hydrophilic lymph (Vogt and Riddiford, 1981; Wojtasek and Leal, 1999), are expressed around the time of eclosion (Gyorgyi et al., 1988).

Insect ORs, a member of a novel family of seven-transmembrane proteins located in the dendrite membrane of OSNs with a reversed membrane topology compared to that of G-protein coupled vertebrate ORs (intracellular N-terminus and extracellular C-terminus) (Clyne et al., 1999; Benton et al., 2006), were first found and identified in *Drosophila melanogaster* (Clyne et al., 1999; Vosshall et al., 1999). In the process of insect olfactory signal transduction, OR and ORCO form a complex of odourant-gated ion channels that play a fundamental role in the conversion of chemical signals to electrical signals (Larsson et al., 2004; Jones et al., 2005; Sato et al., 2008; Smart et al., 2008; Wicher et al., 2008; Butterwick et al., 2018; Fandino et al., 2019).

Ionotropic receptors, belonging to the ionotropic glutamate receptor (iGluR) family of ion channels with three transmembrane domains (M1, M2, and M3), have been shown to be involved in chemosensation (Benton et al., 2009; Croset et al., 2010; Abuin et al., 2011; Bengtsson et al., 2012; Andersson et al., 2013; Tang et al., 2020). Two or three IR genes were co-expressed in an IR-expressing neuron (Benton et al., 2009). IRs are extensively distributed in many insect species, including *D. melanogaster*, *Cydia pomonella*, *Chrysoperla sinica*, *Bactrocera dorsalis*, and *Dendroctonus valens* (Benton et al., 2009; Bengtsson et al., 2012; Gu et al., 2015; Li et al., 2015; Wu et al., 2015), and show relatively high homology across species (Chiu et al., 1999). In insects, IRs are thought to be used for sensing chemicals in the surrounding environment and function during the process of taste perception (Chiu et al., 1999; Benton et al., 2009; Croset et al., 2010).

Sensory neuron membrane proteins, located on dendrite cilia in insects, belong to the CD36 family of two-transmembrane domain membrane proteins (Rogers et al., 2001; Hu et al., 2016). Insect SNMPs can usually be divided into two subfamilies: SNMP1 and SNMP2, while in a recent study, SNMP3 has been found in lepidopteran. SNMP1, with specific expression on pheromone-specific OSNs in the insect antennae, was thought to have a pheromone detection function (Vogt et al., 2009); the function of SNMP2 has not yet been clarified; while is specifically SNMP3 is biased-expressed in the larval midgut, which may be involved in functioning immunity response to virus and bacterial infections the silkworm (Zhang et al., 2020).

*Chilo sacchariphagus* Bojeris, a lepidopteran of the Pyralidae family, is one of the most dangerous pests for sugarcane. Their larvae cause damage by mining the seedlings and stems of sugarcane; this species also harms sorghum, corn and other crops. *C. sacchariphagus* causes great economic losses to the sugar industry every year in China, as well as in South Africa, India, Swaziland, and other countries and regions (Bezuidenhout et al., 2008; Geetha et al., 2010). At present, research on the sugarcane cane borer is mainly focused on identifying resistant varieties, determining the resistance mechanisms of sugarcane and developing biological control techniques (including the utilization of *Trichogramma chilonis* Ishii, pheromones, and pathogenic nematodes) (Nibouche and Tibère, 2010; Nibouche et al., 2012; Sallam et al., 2016). Chemoreception plays an irreplaceable role in the foraging, mating, oviposition and other behaviors of *C. sacchariphagus*, which are vital for its survival in the natural environment. However, few reports have been published on this topic, including on the characterization and function of chemosensory genes and the mechanisms of chemosensory recognition.

In this study, we sequenced and analyzed the *C. sacchariphagus* adult antennal transcriptomes using the Illumina HiSeq^TM^ 4000 platform. Seventy-two chemoreception-related genes were identified in total, including 31 *OBP/PBPs*, 15 *CSPs*, 11 *ORs*, 13 *IRs*, and two *SNMPs*, by analyzing the transcriptome data. Our aim was to identify chemoreception-related genes in this pest insect species, which is destructive to the sugarcane production and sugar industry in China, across Asia and in the Pacific and India. We intend to provide a theory for an improved understanding of how *C. sacchariphagus* recognizes, locates, forages, and mates.

## Materials and Methods

### Insects

The eggs of *C. sacchariphagus*, obtained from a wild field, were reared at 27 ± 1°C with 75 ± 5% relative humidity and a 14 L:10 D photoperiod at Guangdong Engineering Research Center for Pesticide and Fertilizer, Institute of Bioengineering, Guangdong Academy of Sciences, Guangzhou, China. Larvae were reared on an artificial diet under the same conditions. After at least three generations, newly emerged male and female adult *C. sacchariphagus* were chosen as experimental subjects. After pupation, male and female pupae were separated and fed with 10% sugar solution. Antennae of unmated male and female individuals were collected 2 days after eclosion, immediately frozen in liquid nitrogen, and stored at −80°C. Antennae with intact structure were removed using tweezers.

### cDNA Library Construction, Transcriptome Sequencing, Assembly and Functional Annotation

Twenty pairs of antennae and 20 body tissues (without antennae) from male and female of *C. sacchariphagus* were used for RNA extraction. For each sample, total RNA was extracted using TRIzol reagents (Invitrogen, United States) according to the manufacturer’s instructions. RNase-free DNase I (Takara Biotechnology Co., Ltd., Dalian, China) was used to remove contaminating genomic DNA. The quantity and quality of RNA were assessed by agarose gel electrophoresis and on a Bioanalyzer 2100 system (Agilent Technologies, United States). RNA with high purity, concentration and integrity was chosen for cDNA library construction and final Illumina sequencing at Gene Denovo Biotechnology Company (Guangzhou, China). The cDNA was then tested for quality and sequenced on an Illumina HiSeq^TM^ 4000 platform as 150 bp paired-end reads.

The obtained raw reads were processed to remove adapters, primers, low-quality sequences, and ambiguous “N” nucleotides. Then, quality assessment of the clean data was carried out by Q30, and the GC content and sequence duplication level were calculated. Clean data were assembled into contigs using Trinity software and subsequently assembled into transcripts using the De Bruijn graph method. The assembled transcripts were further clustered to form unigenes by using the TGI Clustering Tool (Quackenbush et al., 2001; Pertea et al., 2003).

The annotation of all unigenes was performed by BLASTx against a pooled database containing protein entries from the National Center for Biotechnology Information non-redundant protein (NCBI-NR), Swiss-Prot, Gene Ontology (GO), Clusters of Orthologous Groups (COG), and Kyoto Encyclopedia of Genes and Genomes (KEGG) databases with an *E*-value < 10^–5^. After amino acid sequence prediction, annotation of unigenes was obtained using HMMER software (Eddy, 1998), and Gene Ontology (GO) annotations were determined by Blast2GO. In addition, WEGO (Ye et al., 2006) was utilized to perform GO functional classification and evaluate the distribution of gene functions at the macro level. Unigene functions were also predicted by aligning their sequences with the COG database.

### Phylogenetic Analysis

The amino acid sequence alignment of the candidate chemosensory-related genes of *C. sacchariphagus* was performed using CLUSTALX 2.0 (Larkin et al., 2007). The candidate OBPs, PBPs, CSPs, ORs, IRs, and SNMPs of *C. sacchariphagus* were chosen for phylogenetic analysis along with genes from model organisms Lepidoptera (*Manduca sexta* and *Bombyx mori*), Diptera (*D. melanogaster*), and Hymenoptera (*Apis mellifera*) species. Phylogenetic trees were constructed by the neighbor-joining method, as implemented in MEGA6.0 software. Node support was assessed using a bootstrap procedure with 1000 replicates (Tamura et al., 2013). Phylogenetic trees were colored and arranged using FigTree (Version: 1.4.2).

### Expression Analysis by Real-Time Quantitative PCR (qRT-PCR)

Real-time quantitative PCR (qRT-PCR) was performed to verify the expression of candidate chemosensory genes. Tissue samples were collected from *C. sacchariphagus* adults 2 days after eclosion in three biological replicates, and total RNA was extracted as described above. One microgram of total RNA from the transcriptome samples was subjected to reverse transcription in a total reaction volume of 20 μL according to the manufacturer’s instructions (PrimeScript^TM^ RT Reagent Kit, Takara, Japan) to obtain the first-strand cDNAs. With the manual for the SYBR Green I Master (Roche Diagnostics Ltd., Lewes, United Kingdom), qRT-PCR was processed in 10 μL reaction volumes [1 μL cDNA (2 ng/μL), 5 μL SYBR Green I Master, 0.5 μL/primer, and 3 μL ddH_2_O] on a LightCycler^®^ 480 real-time PCR system (Roche Diagnostics Ltd.) with the following program: denaturation at 95°C for 5 min followed by 40 cycles of 5 s at 95°C, 20 s at 60°C, and 20 s at 72°C. β*-actin* was used as the internal reference gene, and each gene was tested in triplicate. The relative expression levels of the candidate chemosensory genes normalized to the internal control gene were calculated using the 2^–ΔΔ*Ct*^ method (Livak and Schmittgen, 2001).

## Results

### Overview of Transcriptomes

After sequencing and a subsequent quality control process, a total of 16.60 Gb of clean data were obtained from four libraries (CT: antennae of female, CS: body of female, XT: antennae of male, XS: body of male). All the transcriptome libraries generated 231891488 raw reads. A total of 57757438, 61860942, 64297952, and 47525880 clean reads were obtained for CT, CS, XT, and XS, respectively. Then, these clean reads were arranged into 41571, 45477, 41900, and 44065 unigenes for CT, CS, XT, and XS, respectively, with a mean length of 829 bp and N50 length of 1694 bp, using Trinity software (Table 1). The Q30 and GC content of each library were over 93.57% and 46.58%, respectively. Of the unigenes predicted, 24008 (39.96%) had a length between 200 and 300 bp, and 13785 (22.94%) were over 1000 bp in length (Supplementary Figure 1).

**TABLE 1:** Summary of the *C. sacchariphagus* transcriptome.

**Group Name**	**Number**
200–300	24008 (39.96%)
300–500	12445 (20.71%)
500–1000	9848 (16.39%)
1000–2000	7422 (12.35%)
2000+	6361 (10.59%)
Total No. of unigenes	60084
GC percentage (%)	41.33
N50 length (bp)	1694
Maximum unigene length (bp)	23896
Minimum unigene length (bp)	201
Mean length (bp)	829

**FIGURE 1:**
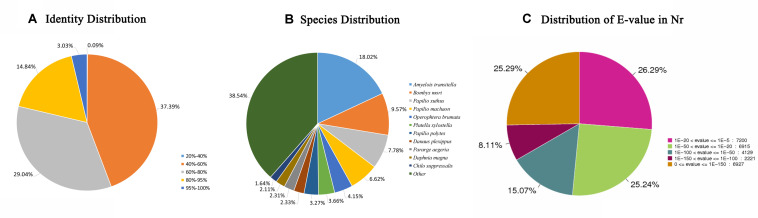
NR classification data. **(A)** The identity distribution of the NR annotation results. **(B)** The species distribution of the NR annotation results. **(C)** The *E*-value distribution of the NR annotation results. NR, non-redundant protein database.

In total, 28330 unigenes (47.15%) were annotated (Table 2). A total of 27392 unigenes (45.59%) were annotated in the NR database, which accounted for the largest proportion of matches, followed by the Swiss-Prot (15150, 25.21%), KOG (12996, 21.63%), and KEGG (11718, 19.50%) databases. The identity levels of the annotation match were >80.00% for 17.87% of the sequences and between 60.00 and 80.00% for 29.04% of the sequences (Figure 1A). According to the NR annotation, 61.64% of the unigenes were annotated with sequences from *Amyelois transitella* (18.02%), *B. mori* (9.57%), *Papilio xuthus* (7.78%), *Papilio machaon* (6.62%), *Operophtera brumata* (4.15%), *Plutella xylostella* (3.66%), *Papilio polytes* (3.27%), *Danaus plexippus* (2.33%), *Pararge aegeria* (2.31%), *Daphnia magna* (2.11%), and *Chilo suppressalis* (1.64%), and 38.54% of the unigenes were annotated with sequences from other species (Figure 1B). Based on the *E* value distribution of the top hits in the NR database, 33.40% and 40.31% of the sequences showed strong (0 ≤ *E*-value ≤ 1.0E^–100^) and moderate (1.0E^–100^ ≤ *E*-value ≤ 1.0E^–20^) homology, respectively (Figure 1C).

**TABLE 2:** Summary of the annotations of the assembled *C. sacchariphagus* unigenes.

Category	Number of unigenses	Percentage (%)
Nr annotation	27392	45.59
SwissProt annotation	15150	25.21
KOG annotation	12996	21.63
KEGG annotation	11718	19.50
Total annotated genes	28330	47.15
Total No. of unigenes	60087	

**FIGURE 2:**
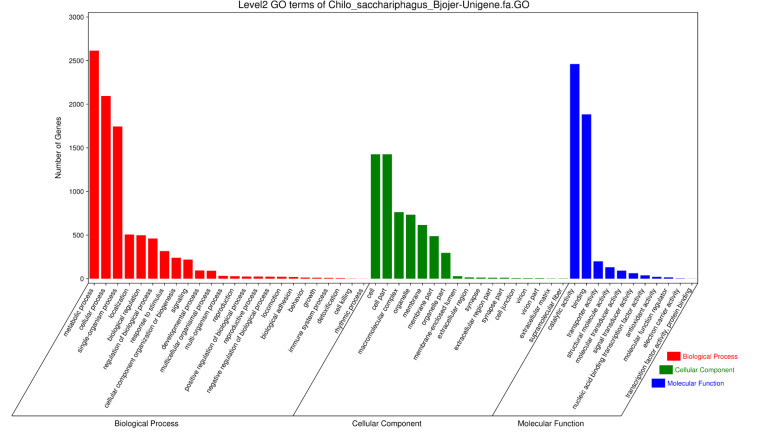
Histogram of Gene Ontology classification of *C. sacchariphagus* unigenes. The right *y*-axis represents the number of genes in each category. The left *y*-axis represents the percentage of a specific category of genes in that main category.

A total of 4662 unigenes were annotated with functional groups classified into 52 subcategories under three main GO categories (“biological process,” “cellular component,” and “molecular function”) via Blast2GO and WEGO software (Figure 2). Among 24 subcategories in the “biological process” category, “metabolic process” and “cellular process” were predominant terms. In the “cellular component” category, “cell part” and “cell” were the most abundant GO terms. Of the 11 subcategories under the “molecular function” category, two contained the largest groups, namely, “catalytic activity” and “binding.”

### Identification of the Candidate Genes Related to Chemoreception

**FIGURE 3:**
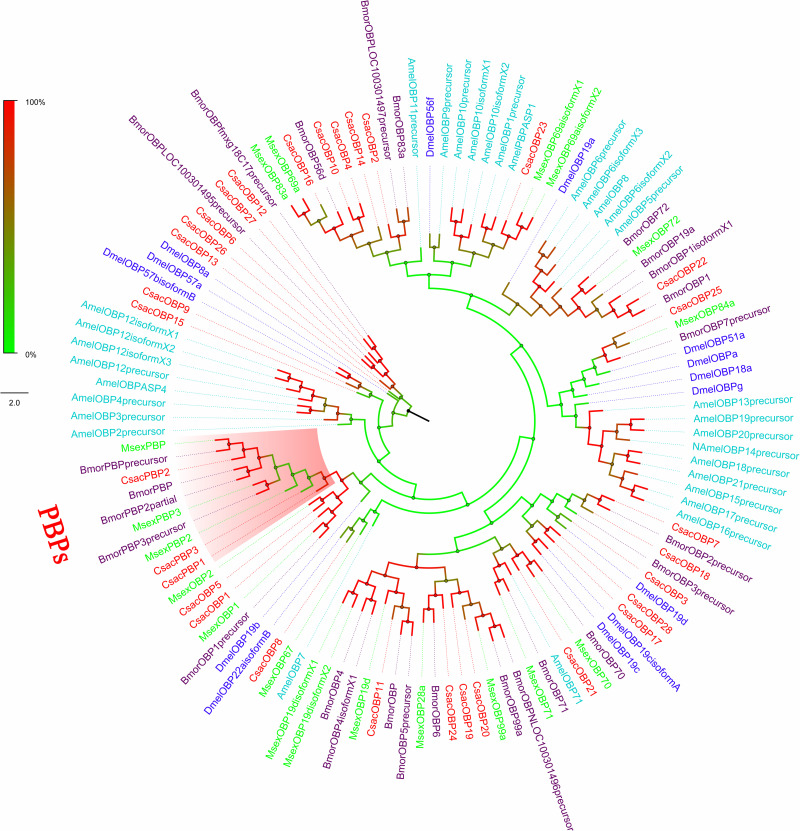
Phylogenetic analysis of putative odourant/phenomenon binding proteins (OBP/PBPs) of *C. sacchariphagus*. The tree was constructed in MEGA6.0 using the neighbor-joining method. Genes from *C. sacchariphagus* are labeled in red. OBP/PBPs from *D. melanogaster* (Diptera) are labeled in dark blue, OBP/PBPs from *B. mori* (Lepidoptera) are labeled in purple, OBP/PBPs from *M. sexta* (Lepidoptera) are labeled in green, and OBP/PBPs from *A. mellifera* (Hymenoptera) are labeled in light blue.

Within this transcriptome, 72 candidate genes related to chemoreception were identified, including 11 *ORs*, 31 *OBPs/PBPs*, 13 *IRs*, 15 *CSPs*, and two *SNMPs*. Twenty-eight different putative sequences encoding odourant binding proteins were identified. Most insect OBPs/PBPs were highly conserved, and 15 candidate OBPs/PBPs (CsacOBP1, CsacOBP2, CsacOBP3, CsacOBP4, CsacOBP5, CsacOBP7, CsacOBP8, CsacOBP10, CsacOBP18, CsacOBP19, CsacOBP20, CsacOBP21, CsacOBP23, CsacOBP26, and CsacPBP2) had an identity higher than 80% with OBPs/PBPs from *Chilo suppressalis*, *Danaus plexippus*, and *Amyelois transitella* (Table 3). According to the prediction, all the CsacOBPs/PBPs possess signal peptides with complete N-termini, except for CsacOBP3, CsacOBP7, CsacOBP12, CsacOBP15, CsacOBP18, CsacOBP25, and CsacPBP3. In the phylogenetic analysis of the OBPs/PBPs in different insect species, CsacOBPs/PBPs were spread across various branches, where they formed five small subgroups together with OBPs/PBPs from other insects (Figure 3). A specific branch consisting of five OBPs from *C. sacchariphagus* (CsacOBP2, CsacOBP4, CsacOBP10, CsacOBP14, and CsacOBP16) was divergent from the OBPs of other insects. The five CsacOBPs have a close relation to OBP83a, OBP56d, and OBPLOC100301497 precursor from *B. mori* and OBP83a and OBP69a from *M. sexta*. CsacOBP6, CsacOBP12, CsacOBP26, and CsacOBP27 formed a small branch that shared a close relationship to OBPfmxg18C7 precursor and OBPLOC100301495 precursor from *B. mori*; in addition, three OBPs from *C. sacchariphagus* (CsacOBP19, CsacOBP20, and CsacOBP24), two OBPs from *M. sexta* (MsexOBP99a and MsexOBP28a) and three OBPs from *B. mori* (BmorOBPLOC100301496 precursor, BmorOBP99a, and BmorOBP6) formed a small subgroup within this branch. However, a specific branch consisting of five closely related genes, *CsacPBP2*, *MsexPBP, BmorPBP* precursor, *BmorPBP*, and *BmorPBP2* partial, was divergent from other OBPs/PBPs.

**TABLE 3:** Unigenes of candidate odorant receptors, ionotropic receptors, odorant binding proteins, sensory neuron membrane proteins, and chemosensory proteins.

Gene name	Unigene reference	Blastx best hit (name)	Species	Length (bp)	ORF (aa)	E-value	Identity (%)	TMD (No.)	Signal peptide
*CsacOBP1*	Unigene0030060	general odorant binding protein 1	*Chilo suppressalis*	675	152	3E-101	85		Yes
*CsacOBP2*	Unigene0030448	general odorant binding protein 1	*Chilo suppressalis*	473	140	8.00E-80	81		Yes
*CsacOBP3*	Unigene0027582	odorant-binding protein 2	*Danaus plexippus*	674	183	3.00E-107	85		No
*CsacOBP4*	Unigene0033446	minus strand odorant-binding protein 2	*Chilo suppressalis*	740	133	2.00E-88	94		Yes
*CsacOBP5*	Unigene0007401	general odorant binding protein 2	*Chilo suppressalis*	731	162	1.00E-103	87		Yes
*CsacOBP6*	Unigene0029763	odorant-binding protein 3	*Cnaphalocrocis medinalis*	865	256	4.00E-92	52		Yes
*CsacOBP7*	Unigene0009252	minus strand odorant-binding protein 3	*Chilo suppressalis*	2079	196	1.00E-114	80		No
*CsacOBP8*	Unigene0032347	odorant-binding protein 4	*Chilo suppressalis*	744	146	2.00E-91	87		Yes
*CsacOBP9*	Unigene0035693	odorant-binding protein 4	*Chilo suppressalis*	999	192	1.00E-78	60		Yes
*CsacOBP10*	Unigene0008372	minus strand odorant-binding protein 5	*Chilo suppressalis*	1116	143	3.00E-83	85		Yes
*CsacOBP11*	Unigene0012927	odorant binding protein 6	*Athetis dissimilis*	496	152	2.00E-42	46		Yes
*CsacOBP12*	Unigene0030417	minus strand odorant-binding protein 7, partial	*Cnaphalocrocis medinalis*	786	241	1.00E-92	56		No
*CsacOBP13*	Unigene0037360	minus strand odorant binding protein 10	*Ostrinia furnacalis*	1460	125	3.00E-83	65		Yes
*CsacOBP14*	Unigene0035330	odorant binding protein 13	*Ostrinia furnacalis*	1275	192	7.00E-84	78		Yes
*CsacOBP15*	Unigene0006183	odorant binding protein 17, partial	*Ostrinia furnacalis*	509	165	2.00E-25	41		No
*CsacOBP16*	Unigene0030117	minus strand odorant-binding protein 18	*Cnaphalocrocis medinalis*	791	138	4.00E-68	75		Yes
*CsacOBP17*	Unigene0006733	minus strand odorant binding protein 20	*Spodoptera litura*	1740	133	1.00E-58	69		Yes
*CsacOBP18*	Unigene0028432	odorant-binding protein 21, partial	*Chilo suppressalis*	683	213	7.00E-98	93		No
*CsacOBP19*	Unigene0039894	minus strand odorant-binding protein 25	*Chilo suppressalis*	614	154	1.00E-94	90		Yes
*CsacOBP20*	Unigene0000195	minus strand odorant-binding protein 29, partial	*Chilo suppressalis*	521	146	1.00E-82	83		Yes
*CsacOBP21*	Unigene0043170	minus strand PREDICTED: general odorant-binding protein 70-like	*Amyelois transitella*	975	184	9.00E-128	96		Yes
*CsacOBP22*	Unigene0005874	PREDICTED: general odorant-binding protein 72-like	*Papilio xuthus*	479	121	1.00E-78	75		Yes
*CsacOBP23*	Unigene0005061	odorant binding protein	*Chilo suppressalis*	1051	133	1.00E-72	83		Yes
*CsacOBP24*	Unigene0032152	odorant binding protein	*Chilo suppressalis*	583	150	1.00E-65	71		Yes
*CsacOBP25*	Unigene0037021	odorant binding protein	*Chilo suppressalis*	862	174	9.00E-68	72		No
*CsacOBP26*	Unigene0038968	odorant-binding protein	*Chilo suppressalis*	893	256	3.00E-165	88		Yes
*CsacOBP27*	Unigene0029475	odorant binding protein	*Eogystia hippophaecolus*	870	239	8.00E-68	44		Yes
*CsacOBP28*	Unigene0042810	minus strand Odorant binding protein	*Operophtera brumata*	651	157	7.00E-87	76		Yes
*CsacPBP1*	Unigene0036519	minus strand pheromone binding protein 1	*Chilo suppressalis*	1123	162	6.00E-87	77		Yes
*CsacPBP2*	Unigene0042820	minus strand pheromone binding protein 2	*Chilo suppressalis*	1766	140	1.00E-89	80		Yes
*CsacPBP3*	Unigene0002457	pheromone binding protein 5	*Ostrinia furnacalis*	1604	165	1.00E-46	45		No
*CsacCSP1*	Unigene0012225	chemosensory protein 3	*Agrotis ipsilon*	524	120	5.00E-34	48		Yes
*CsacCSP2*	Unigene0004638	chemosensory protein 4	*Ostrinia furnacalis*	1431	129	7.00E-73	82		Yes
*CsacCSP3*	Unigene0007810	chemosensory protein 6	*Conogethes punctiferalis*	699	123	8.00E-54	63		Yes
*CsacCSP4*	Unigene0029070	minus strand chemosensory protein 10	*Ostrinia furnacalis*	425	121	2.00E-43	58		Yes
*CsacCSP5*	Unigene0001797	chemosensory protein 14	*Spodoptera exigua*	2497	333	5.00E-107	58		Yes
*CsacCSP6*	Unigene0007266	chemosensory protein 16	*Ostrinia furnacalis*	455	118	2.00E-36	50		Yes
*CsacCSP7*	Unigene0031023	chemosensory protein 18	*Ostrinia furnacalis*	547	105	2.00E-51	78		Yes
*CsacCSP8*	Unigene0035672	chemosensory protein 36	*Cnaphalocrocis medinalis*	860	121	6.00E-51	69		Yes
*CsacCSP9*	Unigene0002397	chemosensory protein	*Chilo suppressalis*	523	121	3.00E-74	86		Yes
*CsacCSP10*	Unigene0002847	chemosensory protein, partial	*Chilo suppressalis*	1027	120	7.00E-72	89		Yes
*CsacCSP11*	Unigene0035354	minus strand chemosensory protein, partial	*Chilo suppressalis*	1620	167	2.00E-68	71		Yes
*CsacCSP12*	Unigene0001848	minus strand chemosensory protein	*Cnaphalocrocis medinalis*	1001	190	2.00E-57	59		Yes
*CsacCSP13*	Unigene0004808	minus strand chemosensory protein	*Cnaphalocrocis medinalis*	606	130	3.00E-69	75		Yes
*CsacCSP14*	Unigene0041621	chemosensory protein	*Cnaphalocrocis medinalis*	2154	121	4.00E-64	86		Yes
*CsacCSP15*	Unigene0033697	chemosensory protein	*Eogystia hippophaecolus*	1365	145	1.00E-51	64		Yes
*CsacORCO*	Unigene0033699	minus strand olfactory receptor 2	*Chilo suppressalis*	1664	342	0	96	6	
*CsacOR1*	Unigene0007696	odorant receptor 13a-like	*Plutella xylostella*	1651	454	2.00E-129	45	6	
*CsacOR2*	Unigene0011933	odorant receptor 50, partial	*Manduca sexta*	1174	365	5.00E-127	50	4	
*CsacOR3*	Unigene0026875	olfactory receptor 43, partial	*Cnaphalocrocis medinalis*	900	272	2.00E-90	47	3	
*CsacOR4*	Unigene0028620	minus strand odorant receptor	*Eogystia hippophaecolus*	1552	401	1.00E-136	49	6	
*CsacOR5*	Unigene0033533	minus strand odorant receptor 13a-like	*Plutella xylostella*	894	292	3.00E-61	38	4	
*CsacOR6*	Unigene0037945	minus strand odorant receptor 60	*Athetis dissimilis*	1428	229	2.00E-86	71	3	
*CsacOR7*	Unigene0023407	odorant receptor	*Eogystia hippophaecolus*	448	127	6.00E-37	43	2	
*CsacOR8*	Unigene0057813	odorant receptor 14, partial	*Cnaphalocrocis medinalis*	709	160	5.00E-75	68	2	
*CsacOR9*	Unigene0010994	minus strand olfactory receptor 40	*Cnaphalocrocis medinalis*	413	122	9.00E-65	68	2	
*CsacOR10*	Unigene0028643	olfactory receptor 56	*Bombyx mori*	520	107	2.00E-56	67	2	
*CsacIR1*	Unigene0005443	ionotropic receptor 1, partial	*Cnaphalocrocis medinalis*	781	247	3.00E-175	94	2	
*CsacIR2*	Unigene0030792	minus strand ionotropic receptor 1	*Heliconius melpomene rosina*	923	137	3.00E-73	67	0	
*CsacIR3*	Unigene0026968	ionotropic receptor	*Ostrinia furnacalis*	1003	268	2.00E-169	85	1	
*CsacIR4*	Unigene0038631	ionotropic receptor	*Ostrinia furnacalis*	2004	547	0	74	3	
*CsacIR5*	Unigene0040849	ionotropic receptor	*Ostrinia furnacalis*	2884	836	0	95	3	
*CsacIR6*	Unigene0045750	minus strand ionotropic receptor, partial	*Ostrinia furnacalis*	1469	233	2.00E-129	78	1	
*CsacIR7*	Unigene0019248	minus strand ionotropic receptor, partial	*Ostrinia furnacalis*	935	280	3.00E-151	72	3	
*CsacIR8*	Unigene0027705	ionotropic receptor, partial	*Dendrolimus kikuchii*	747	100	2.00E-80	55	0	
*CsacIR9*	Unigene0025240	ionotropic receptor	*Ostrinia furnacalis*	734	232	3.00E-102	65	0	
*CsacIR10*	Unigene0011763	ionotropic receptor	*Ostrinia furnacalis*	478	110	7.00E-78	72	0	
*CsacIR11*	Unigene0018788	ionotropic receptor	*Ostrinia furnacalis*	465	110	2.00E-61	74	0	
*CsacIR12*	Unigene0005556	ionotropic receptor 21a.1, partial	*Cnaphalocrocis medinalis*	415	127	2.00E-44	62	0	
*CsacIR13*	Unigene0019250	ionotropic receptor, partial	*Ostrinia furnacalis*	395	108	2.00E-23	37	0	
*CsacSNMP1*	Unigene0013065	minus strand sensory neuron membrane protein 1	*Chilo suppressalis*	1852	526	0	83	2	
*CsacSNMP2*	Unigene0007127	minus strand sensory neuron membrane protein 2	*Chilo suppressalis*	1896	519	0	82	1	

*The putative N-terminal signal peptides and most likely cleavage sites were predicted using SignalP V3.0 (http://www.cbs.dtu.dk/services/SignalP/). The transmembrane helices in the ORs, IRs, and SNMPs were predicted using TMHMM Server v. 2.0 (http://www.cbs.dtu.dk/services/TMHMM/).*

**FIGURE 4:**
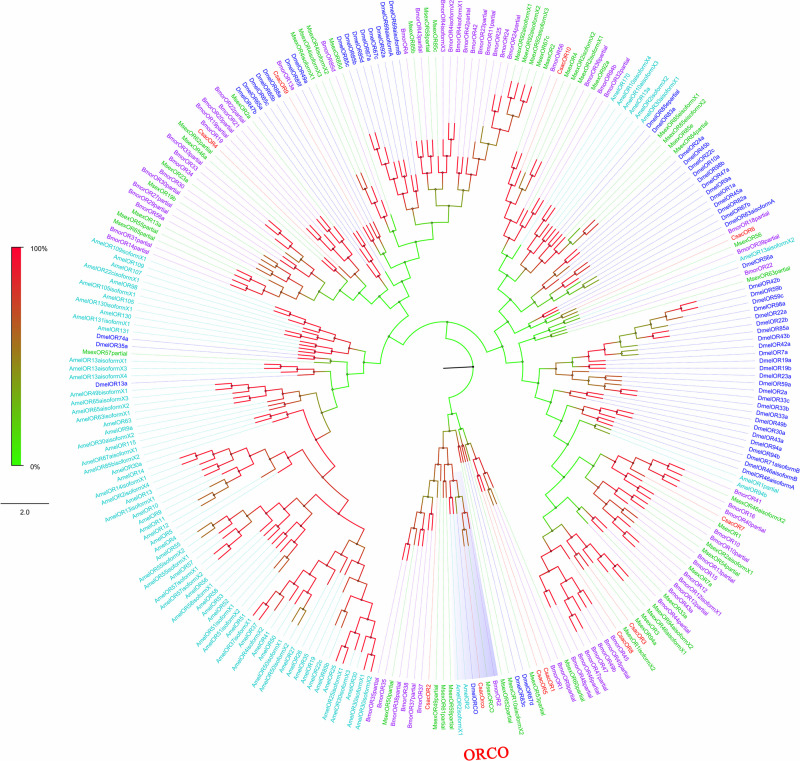
Phylogenetic analysis of putative olfactory receptors (ORs) of *C. sacchariphagus*. The tree was constructed in MEGA6.0 using the neighbor-joining method. Genes from *C. sacchariphagus* are labeled in red. ORs from *D. melanogaster* (Diptera) are labeled in dark blue, ORs from *B. mori* (Lepidoptera) are labeled in purple, ORs from *M. sexta* (Lepidoptera) are labeled in green, and ORs from *A. mellifera* (Hymenoptera) are labeled in light blue.

Among the 11 candidate *ORs*, four were of short length (no more than 100 amino acids), and the remaining seven possessed a deduced protein longer than 200 amino acids (Table 3). From the prediction, three sequences (*CsacORCO*, *CsacOR1*, and *CsacOR4*) were full-length *OR* genes with intact open reading frames with a general length of 1500 bp and 5–7 transmembrane domains, which are characteristic of typical insect ORs. Compared with OBPs, the results of BLASTx revealed that the identity of these candidate ORs with known insect ORs was relatively low. Only one candidate OR (CsacORCO) had an identity higher than 80% (96%) with its closest match, while the identities of the remaining ORs ranged from 38 to 71%. Two ORs, CsacOR1 and CsacOR5, formed a small branch that was closely related to BmorOR1 and BmorOR9 from *B. mori* and MsexOR60 from *M. sexta*, and these ORs formed a distinct subgroup (Figure 4). Most of the splits in the tree were supported by bootstrap values, and only a few splits were unreliable.

**FIGURE 5:**
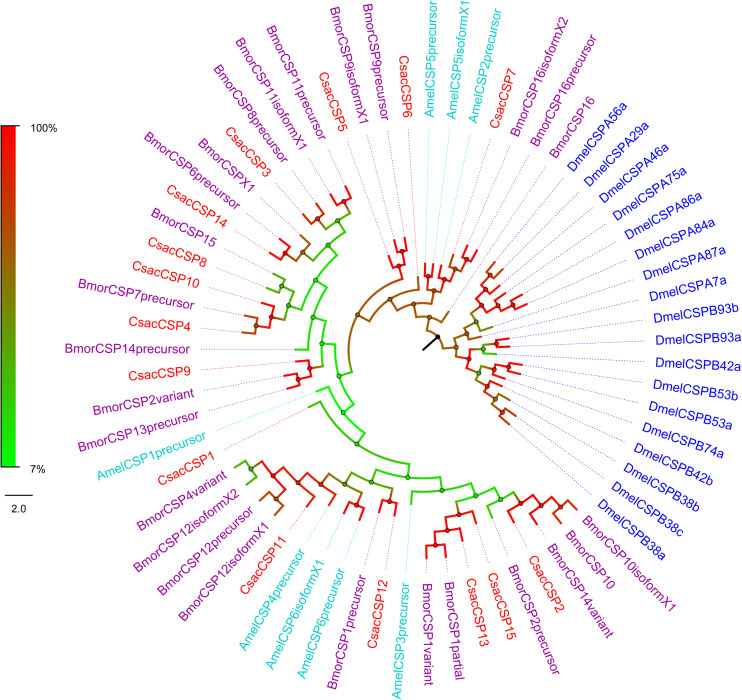
Phylogenetic analysis of putative chemosensory proteins (CSPs) of *C. sacchariphagus*. The tree was constructed in MEGA6.0 using the neighbor-joining method. Genes from *C. sacchariphagus* are labeled in red. CSPs from *D. melanogaster* (Diptera) are labeled in dark blue, CSPs from *B. mori* (Lepidoptera) are labeled in purple, CSPs from *M. sexta* (Lepidoptera) are labeled in green, and CSPs from *A. mellifera* (Hymenoptera) are labeled in light blue.

Bioinformatic analysis led to the identification of 15 different sequences encoding candidate *CsacCSPs*. Due to their complete N-termini, all the sequences had signal peptides. The identity of the 15 CsacCSPs ranged from 48 to 89% (Table 3). Neighbor-joining tree analysis showed that CsacCSP13 and CsacCSP15 formed a specific branch that was close to BmorCSP1 and BmorCSP1 variant from *B. mori*. Additionally, a specific branch consisting of two CSPs from *C. sacchariphagus* (CsacCSP4 and CsacCSP10) was divergent from the CSPs of other insects, and the two CsacCSPs have a close relationship to CSP7 precursor from *B. mori* (Figure 5).

**FIGURE 6:**
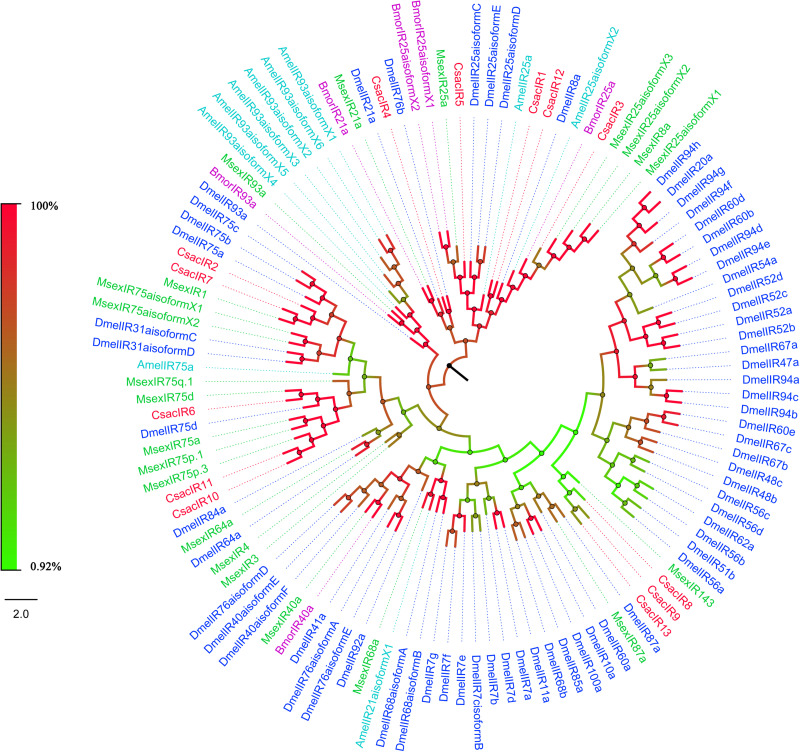
Phylogenetic analysis of putative ionotropic receptors (IRs) of *C. sacchariphagus*. The tree was constructed in MEGA6.0 using the neighbor-joining method. Genes from *C. sacchariphagus* are labeled in red. IRs from *D. melanogaster* (Diptera) are labeled in dark blue, IRs from *B. mori* (Lepidoptera) are labeled in purple, IRs from *M. sexta* (Lepidoptera) are labeled in green, and IRs from *A. mellifera* (Hymenoptera) are labeled in light blue.

The putative *IR* genes in the *C. sacchariphagus* transcriptome were represented according to their similarity to known insect *IRs*. Bioinformatic analysis led to the identification of 13 candidate *IRs*, of which eight candidate IRs had higher than 70% identity with known insect IRs, and only two had identities lower than 60%. Compared with general insect IRs, which have three transmembrane domains, three IR candidates in *C. sacchariphagus* (CsacIR4, CsacIR5, and CsacIR7) were predicted to have three transmembrane domains by TMHMM2.0 (Table 3). In the phylogenetic analysis, CsacIR2, CsacIR7, and IRs from *M. sexta* (MsexIR1) and *D. melanogaster* (DmelIR75a, DmelIR75b, and DmelIR75c) formed a distinct subgroup, while CsacIR6, CsacIR10, and CsacIR11 formed a branch that shared a close relation to IR75d from *D. melanogaster* and IR75a, IR75p.1, and IR75p.3 from *M. sexta*; additionally, CsacIR1, CsacIR3, and CsacIR12 formed a specific branch consisting of DmelIR8a, AmelIR25a MsexIR8a, MsexIR25a, and BmorIR25a with their positions in phylogenetic tree and strong bootstrap support (Figure 6).

**FIGURE 7:**
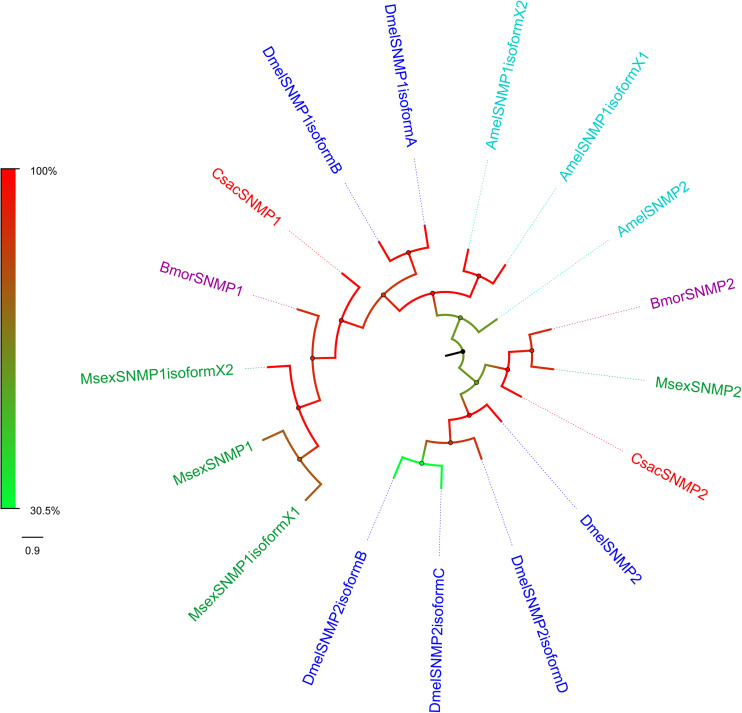
Phylogenetic analysis of putative sensory neuron membrane proteins (SNMPs) of *C. sacchariphagus*. The tree was constructed in MEGA6.0 using the neighbor-joining method. Genes from *C. sacchariphagus* are labeled in red. SNMPs from *D. melanogaster* (Diptera) are labeled in dark blue, SNMPs from *B. mori* (Lepidoptera) are labeled in purple, SNMPs from *M. sexta* (Lepidoptera) are labeled in green, and SNMPs from *A. mellifera* (Hymenoptera) are labeled in light blue.

Sensory neuron membrane proteins were identified in pheromone-sensitive neurons in Lepidopteran insects and are thought to function in the process of pheromone recognition (Rogers et al., 2001). Two *SNMPs* (*CsacSNMP1* and *CsacSNMP2*) were identified in our transcriptome. Both of them all have an identity of greater than 80% with SNMPs of *Chilo suppressalis* (Table 3). According to the phylogenetic analysis, both *C. sacchariphagus* candidate SNMPs clustered with their SNMP orthologs into separate subclades (Figure 7), among which CsacSNMP1, BmorSNMP1, and MsexSNMP1 formed a specific branch and CsacSNMP2 and SNMP2 from *B. mori* and *M. sexta* shared a close relationship.

### Tissue- and Sex-Specific Expression of Candidate Chemosensory Genes

**FIGURE 8:**
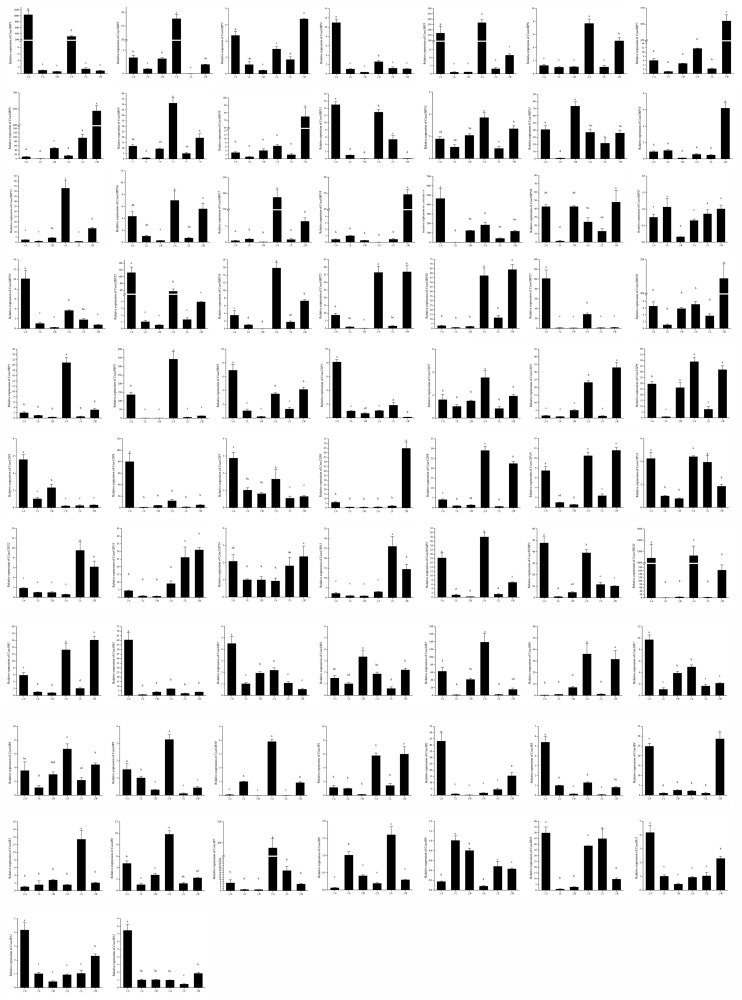
Expression patterns of putative odourant/phenomenon binding proteins (OBP/PBPs), chemosensory proteins (CSPs), sensory neuron membrane proteins (SNMPs), odourant receptors (ORs), and ionotropic receptors (IRs) in the different tissues of *C. sacchariphagus* as determined using RT-qPCR. ♀A, female antennae; ♂A, male antennae; ♀L, female legs; ♂L, male legs; ♀B, female body (without antennae and legs); ♂B, male body (without antennae and legs). Error bars indicate SEMs from the analysis of three replicates (*P* < 0.05). The lower case letters indicate that there are significant differences between the data.

To validate and analyze the expression profiles of candidate chemosensory genes in different organs and tissues between male and female *C. sacchariphagus*, all candidate chemosensory genes encoding OBPs/PBPs, CSPs, ORs, IRs, and SNMPs were subjected to RT-qPCR with specific primers (Supplementary Table 1). The expression difference of chemosensory genes from transcriptome data was shown in heatmap (Supplementary Figure 2). The expression patterns of the 72 chemosensory genes were basically consistent with the FPKM values, and the data are presented as log_2_ values of fold changes in gene expression. According to the RT-qPCR results, a large number of chemosensory genes were antenna-predominant and showed different expression levels between males and females (*P* < 0.05). Among these genes, the expression levels of genes (*CsacOBP2/5/6/9/12/15/17/24/25/26*, *CsacPBP1/2*, *CSP2/3/4/9/10*, *CsacOR1/5/6/8/9/10*, *IR1/6/7*, and *CsacSNMP1*) were higher in male antennae than that in female antennae (Figure 8), whereas the opposite occurred was observed for the other genes expression (*CsacOBP1/3/4/11/19/22/23/27*, *CsacPBP3*, *CsacCSP1/5/6/7*, *CsacOR2/3/7*, *CsacIR2/3/4/11/12*, and *CsacSNMP2*) (Figure 8). In addition, some genes (*CsacOBP3/7/8/10/13/14/18/20/25/26/28*, *CsacCSP3/4/8/9/10/11/12/13/15*, *CsacOR1/4/6*, and *CsacIR1/4/8/9/10*) had a high expression in bodies (excluding antennae and legs) or legs (Figure 8).

## Discussion

In this study, the transcriptome of the pest *C. sacchariphagus* was analyzed using Illumina HiSeq^TM^ 4000 technology. We obtained 16.60 GB of clean data that was assembled into 60084 unigenes with a mean length of 829 bp and N50 length of 1694 bp. There were 60.67% unigenes with a length <500 bp after assembly, possibly due to the short-length sequencing capacity of Illumina sequencing. Among the 60084 unigenes, 28330 unigenes were annotated, and 52.85% of unigenes had no significant match in any of the databases searched. This phenomenon may be caused by the lack of genomic and transcriptomic information for this moth in the databases. This antennal and body transcriptome sequencing provides a dataset of chemosensory genes, including 28 *OBPs*, three *PBPs*, 15 *CSPs*, 11 *ORs*, 13 *IRs*, and two *SNMPs*.

Odourant/pheromone binding proteins interact with semiochemicals, hormones or other biologically active chemicals that enter the body through pores and then transport them to ORs located on the membranes of olfactory receptor neurons (Pelosi and Maida, 1995; Vogt, 1995; Kaissling, 1998). Fewer *OBPs/PBPs* were identified in this transcriptome of *C. sacchariphagus* (31) than in *B. mori* (44) or *D. melanogaster* (51) (Hekmat-Scafe et al., 2002; Gong et al., 2007). The difference in the number of *OBPs* might be related to the sequencing method, depth, the process of sample preparation or evolutionary differences across different species. These results are comparable to those reported for the transcriptomes of *Spodoptera littoralis* (33), *Spodoptera exigua* (34), and *Helicoverpa armigera* (26) (Liu N. Y. et al., 2012; Liu Y. et al., 2012; Poivet et al., 2013; Liu et al., 2015; Walker et al., 2019). This suggests that *C. sacchariphagus OBPs* show conservation in gene numbers. Some OBPs are conserved and have orthologous relationships with counterparts from other insects. Insect *OBPs/PBPs*, mainly expressed in the antennae, are considered to have an olfactory function. Analysis of *OBP/PBPs* expression profiles in different organs and tissues could reveal their likely functions. qRT-PCR results showed that 22 *CsacOBPs/PBPs* displayed antenna-enriched expression, indicating that these genes may play critical roles in the process of olfactory reception. Among these genes, 13 (*CsacOBP2/5/6/9/12/15/17/24/25/26/27* and *CsacPBP1/2*) were mainly expressed in male antennae, suggesting that these genes may encode proteins involved in sex-specific behaviors, including selectively sensing and transporting sex pheromones released by females in the process of molecular recognition and searching for suitable mates (Gu et al., 2013; Jin et al., 2014; Chang et al., 2015; Zhu et al., 2016, 2019). Ten genes (*CsacOBP7/8/10/13/14/16/18/20/21/28*) without significant differences in expression levels between males and females may function as general odourant detectors rather than in pheromone recognition (Li et al., 2008; Pelletier and Leal, 2009; He et al., 2019a). Some genes (*CsacOBP1/3/4/11/19/22/23/27*) showed female antenna-biased expression, indicating that those OBPs may help to locate oviposition sites by recognizing chemicals from hosts, a model that is supported by previous studies of *Pieris rapae* (Renwick et al., 1992; Sato et al., 1999; Li et al., 2020).

Fifteen *CSPs* were identified in transcriptome sequencing. This number is almost equal to the number of *CSPs* in *H. armigera* (18), *Heliothis assulta* (17), *S. littoralis* (21), *B. mori* (20), and *S. exigua* (20) but much higher than that of *D. melanogaster* (4) (Wanner et al., 2004; Gong et al., 2007; Zhou et al., 2010; Poivet et al., 2013; Leitch et al., 2015; Liu et al., 2015; Zhang et al., 2015; Walker et al., 2019), indicating that the numbers of *CSP* genes differ among different species. *CSPs* exist in insect chemosensory and non-chemosensory organs and tissues, including antennae, legs, pheromone glands, and wings (Picimbon et al., 2001; Ban et al., 2003; Dani et al., 2011; Liu N. Y. et al., 2012; Wei et al., 2017). In our study, 10 *CsacCSPs* were significantly expressed in the antennae, and these CSPs might be thought to participate in general odourant recognition and perception (Pelosi et al., 2014; Jia et al., 2018). Four *CSPs* showed high expression in legs and might be associated with gustatory behaviors, such as detecting non-volatile chemicals (Jia et al., 2020).

In the qRT-PCR analysis, some identified *CsacOBPs* and *CsacCSPs* displayed high expression in male bodies, and we speculated that these genes are likely to be involved in different functions in non-sensory organs and tissues of the insect body. Some OBPs and CSPs in male insect seminal fluid might be related to binding and releasing pheromones. In *D. melanogaster*, OBPs were found to be components of the seminal fluid (Takemori and Yamamoto, 2009); LmigCSP91 was identified to have a high expression in reproductive organs in male *Locusta migratoria* and possessed a good affinity to a kind of pheromone that is produced in the same reproductive organs (Ban et al., 2013; Zhou et al., 2013). Some OBPs are male specific and could be transferred into female bodies during the process of mating, indicating that these OBPs might function in sperm–egg communication (Findlay et al., 2008; Takemori and Yamamoto, 2009; Prokupek et al., 2010). In addition, CSPs are involved in releasing some molecules in male glands; for example, a CSP was found in large quantities in the ejaculatory apparatus, which secretes the male pheromone vaccenyl acetate (Dyanov and Dzitoeva, 1995).

Odourant receptors act as the most critical and determinate roles in insect peripheral olfactory reception (Dani et al., 2011; Leal, 2013). Eleven *ORs* were identified in our research, and this number was lower than the numbers identified in *B. mori* (72) (Gong et al., 2009), *M. sexta* (73) (Koenig et al., 2015), *H. armigera* (84) (Pearce et al., 2017), *Heliconius melpomene* (74) (Dasmahapatra et al., 2012), *D. melanogaster* (62) (Clyne et al., 1999; Gao and Chess, 1999; Robertson et al., 2003), *Laodelphax striatellus* (133) (He et al., 2020), *Sogatella furcifera* (135) (He et al., 2018), and *A. mellifera* (170) (Robertson and Wanner, 2006), suggesting that different sequencing methods and depths may affect the outcome of studies; the lack of genomic and transcriptomic information in the databases may influence the annotation results for *C. sacchariphagus*, and some ORs expressed at low levels may be difficult to detect (Li et al., 2015; Wang et al., 2017). In the neighbor-joining tree of ORs, CsacOR1 and CsacOR5 are orthologs of BmorOR1; CsacOR4 is the ortholog of BmorOR19; and CsacOR10 clustered close to BmorOR56. In *B. mori*, OR1 is the receptor of the pheromone bombykol; OR19 can sense linalool, which is related to selection of spawning environment; and OR56, specific and highly sensitive to *cis-*jasmone, is involved in the sensing of odor molecules released by plants and signal transduction (Wanner et al., 2007; Anderson et al., 2009; Tanaka et al., 2009). The qRT-PCR results showed that *CsacOR1/5/10* were highly expressed in the male antennae, suggesting that they are highly specifically involved in the detection of sex pheromones, while *CsacOR4* has a higher expression in the female body than in the male body, indicating that it is likely involved in the regulation of female-specific behaviors, such as the localization of oviposition sites and oviposition (Xu et al., 2020). The expression of *CsacORCO*, which was highly conserved in the OR tree, was significantly antenna-specific. The different expression levels of the *ORs* in different organs and tissues and different sexes suggested that they might perform different functions, which should be further studied in the future.

Thirteen IR genes were identified in this study from *C. sacchariphagus.* The number is similar to that of *B. mori* (18), *H. armigera* (12), and *S. littoralis* (12) (Croset et al., 2010; Olivier et al., 2011; Liu Y. et al., 2012). Most CsacIRs were clustered with orthologs in *D. melanogaster*, *M. sexta*, *B. mori*, and *A. mellifera*, indicating that IRs are relatively conserved in different insect species. In *D. melanogaster*, IR84a/8a, IR76b/IR41a, IR75a/IR8a, IR64a/IR8a have been reported to sense phenylacetaldehyde and phenylacetic acid, polyamines, acetic acid, and other acids, respectively (Ai et al., 2010; Grosjean et al., 2011; Hussain et al., 2016; Prieto-Godino et al., 2016). And in *M. sexta*, MsexIR8a has been shown the function of sensing carboxylic acids 3-methylpentanoic acid and hexanoic acid (Zhang et al., 2019). In addition, DmelIR21a/IR25a have been reported to be sensitive to cool temperatures (Ni et al., 2016). The CsacIR genes showed high sequence similarity to these functionally characterized DmelIRs, indicating that they may have similar functions.

In insects, *SNMP1* is usually expressed in pheromone-sensitive OSNs and is important for pheromone perception (Jin et al., 2008; Nichols and Vogt, 2008; Vogt et al., 2009; Gomez-Diaz et al., 2016). However, *SNMP2* functions remain unclear. In the present study, two *SNMPs* were identified in *C. sacchariphagus.* Both were conserved with respect to other holometabolous insect species. They exhibited a clear antenna-predominant expression, suggesting that *CsacSNMP1* may be associated with pheromone reception.

In conclusion, 72 candidate chemosensory protein genes (31 *OBP/PBPs*, 15 *CSPs*, 11 *ORs*, 13 *IRs*, and two *SNMPs*) were first identified via transcriptome sequencing analysis in *C. sacchariphagus*, which is an important agricultural pest. This study will not only serve as a valuable resource for future research on the chemosensory system of *C. sacchariphagus* and other lepidopteran species but also contribute to the development of creative and sustainable pest management strategies involving interference with olfaction.

## Data Availability Statement

The datasets presented in this study can be found in online repositories. The names of the repository/repositories and accession number(s) can be found in the article/Supplementary Material.

## Author Contributions

JL, HL, JY, YA, and HW conceived, coordinated, and designed the research. YM, JL, and DS assembled and analyzed the transcriptome datasets. JL and HL performed experiments. JL, JY, YA, and HW drafted the manuscript. All authors read and approved the final manuscript.

## Conflict of Interest

The authors declare that the research was conducted in the absence of any commercial or financial relationships that could be construed as a potential conflict of interest.

## Supplementary Material

Supplementary Figure 1 Length distribution of unigenes in transcriptomes of *Chilo sacchariphagus*.

Supplementary Figure 2 The expression difference of chemosensory genes from transcriptome data.

Supplementary Table 1 Primers used in qRT-PCR.

## Abbreviations

OR: odorant receptor
IR: ionotropic receptor
PBP: pheromone binding protein
OBP: odorant binding protein
CSP: chemosensory protein
SNMP: sensory neuron membrane protein
GO: gene ontology
FPKM: fragments per kb per million fragments
FDR: false discovery rate.
