# Supplementary figures and images for: Transcriptome Characterization and Expression Analysis of Chemosensory Genes in Chilo sacchariphagus (Lepidoptera Crambidae), a Key Pest of Sugarcane
Source: Front Physiol. 2021 Mar 5;12:636353. doi: 10.3389/fphys.2021.636353 (PMC7982955; doi:10.3389/fphys.2021.636353)

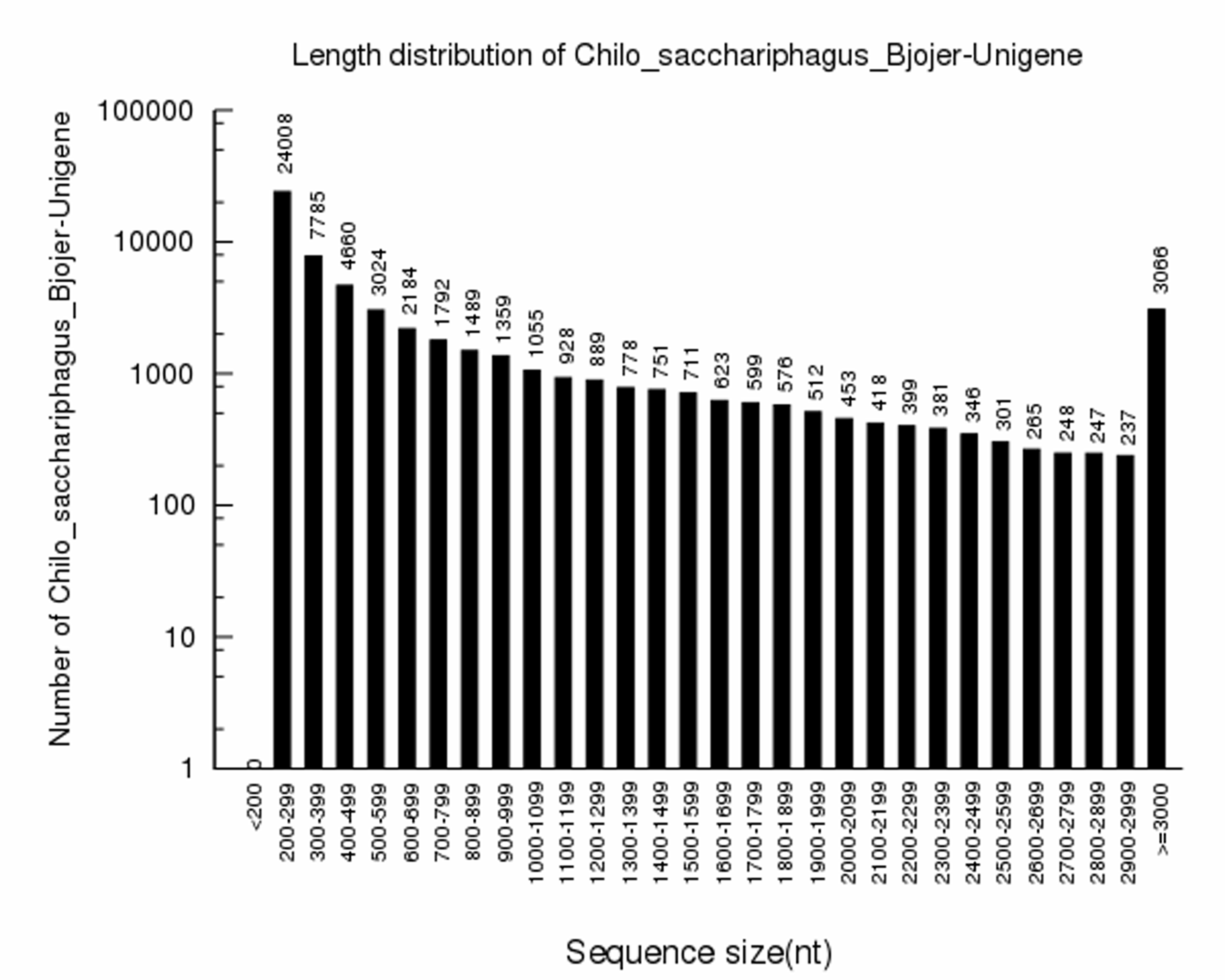

Supplement: Supplementary Figure 1 — Length distribution of unigenes in transcriptomes of Chilo sacchariphagus. [file Image_1.TIF]

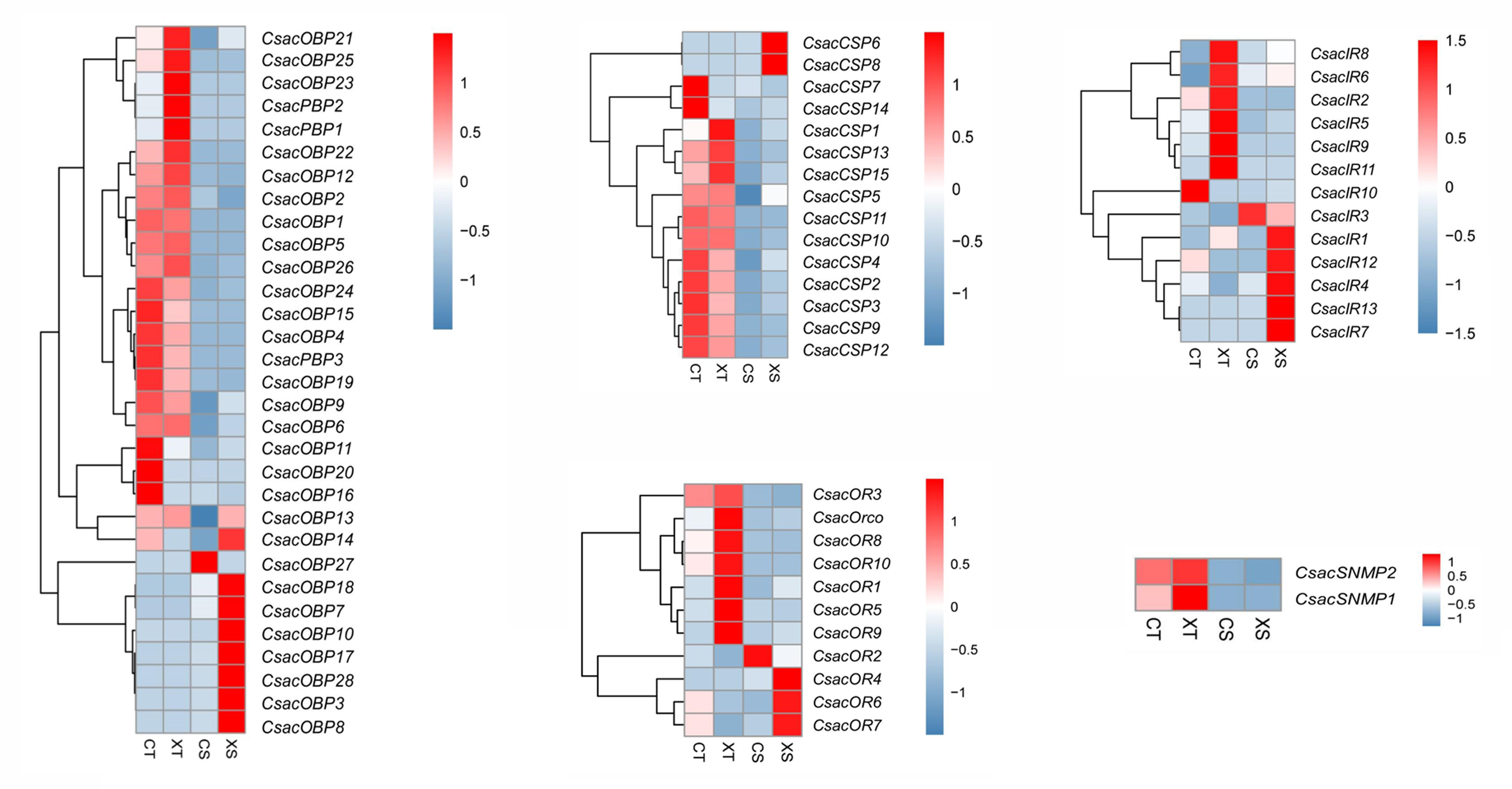

Supplement: Supplementary Figure 2 — The expression difference of chemosensory genes from transcriptome data. [file Image_2.TIF]
