# Supplementary material for: Transcriptome Characterization and Expression Analysis of Chemosensory Genes in Chilo sacchariphagus (Lepidoptera Crambidae), a Key Pest of Sugarcane
Source: Front Physiol. 2021 Mar 5;12:636353. doi: 10.3389/fphys.2021.636353 (PMC7982955; doi:10.3389/fphys.2021.636353)
Supplement: Supplementary Table 1 — Primers used in qRT-PCR. [file Table_1.docx]

Supplementary Table

Primers used qRT-PCR

| Gene | Primer sequences (5’-3’) | S/AS |
| --- | --- | --- |
| *actin* | CAATCCTAAAGCCAACAGA | S |
|  | GCGTAGCCCTCGTAGAT | AS |
| *CsacOBP1* | ATCACGAGGAGGACCACT | S |
|  | CTCAGCGAGCAGCATTT | AS |
| *CsacOBP2* | GCAAGTGGGAAATCAT | S |
|  | TCCTTTAGGGAACACG | AS |
| *CsacOBP3* | GTGTTTCGCTGCCTGTA | S |
|  | TGCCGTCAATGCTGTC | AS |
| *CsacOBP4* | TTTTATCGTGTTGGCTGTC | S |
|  | CCACTTTAGTCTCCGTCAG | AS |
| *CsacOBP5* | GATGAGTGCCGTGAAGAG | S |
|  | CTGATGGATGCGAGTGTC | AS |
| *CsacOBP6* | CGCCGCCAACAACCTCT | S |
|  | CATCGCCCGTTCCATCA | AS |
| *CsacOBP7* | TTCGCGTGTTGTTTGGT | S |
|  | TGGAGGCTTTCACTTGG | AS |
| *CsacOBP8* | ATGTTTCGTTTGGTGTTCTT | S |
|  | AGGGTTGGAGCAATCAGT | AS |
| *CsacOBP9* | ACGCAGGTGGCGGAGTA | S |
|  | ACGCTGGGCAGTTGAGG | AS |
| *CsacOBP10* | CTCAAGATGGGAAATTGGA | S |
|  | CAGCAACTTCTTTGGGTAA | AS |
| *CsacOBP11* | AAAATGCCAGACAATGAGA | S |
|  | CATCCACGAACTTGAAACC | AS |
| *CsacOBP12* | GGCTTCTAAATGTGCTGC | S |
|  | CACTATCAACGGGTCCAA | AS |
| *CsacOBP13* | AAATGAGGTGGAAGAGGC | S |
|  | ATGGTGCTGTCGTTGAAGT | AS |
| *CsacOBP14* | CATCCAGCGACAACAGG | S |
|  | CCAACTTCGGGTCCACT | AS |
| *CsacOBP15* | CTACCCGATTCACCTAT | S |
|  | GTTCTTTGATTGCCTTT | AS |
| *CsacOBP16* | GTCAAGCAAACGGGAGT | S |
|  | GGAAGAAGCAAAGGGTG | AS |
| *CsacOBP17* | ATCGCTGTGATGTATGCT | S |
|  | GGTTCATCCGACTCCTG | AS |
| *CsacOBP18* | AGTACAGGCGAACGACAA | S |
|  | TTCTTCATCAACCGAGTATCT | AS |
| *CsacOBP19* | CTGTCCACTGGCATAGA | S |
|  | AAAGCATTTGGCATC | AS |
| *CsacOBP20* | CGCTCAATAACGAACAA | S |
|  | TAAGGACGCAAGCACTA | AS |
| *CsacOBP21* | GAAAGTGAAAGCGGTGGA | S |
|  | GAAGTGGTCGTGGTGGC | AS |
| *CsacOBP22* | TTGAATTACGAGGCTGTCC | S |
|  | TTTCTTCCCAACATCTTTAC | AS |
| *CsacOBP23* | ATGATGACGGACGGAGAA | S |
|  | ACCTGCGTGAGGAAAGC | AS |
| *CsacOBP24* | GCTGCGGCTCTAAG | S |
|  | TTCGGACACCTCAAA | AS |
| *CsacOBP25* | GCTTACTTGGAGGCATTG | S |
|  | GCGGTCACATCTGGGT | AS |
| *CsacOBP26* | CCGTACCATCAGAAAGA | S |
|  | TCAAATCCACCTCCATA | AS |
| *CsacOBP27* | GCGTTACTTCCTTTGATG | S |
|  | GCGTCGTGGACTGTTAT | AS |
| *CsacOBP28* | ATTCTTCTCGCACAGC | S |
|  | CATTATGCCAAACTTCT | AS |
| *CsacPBP1* | GCGGTGATGAAGTCG | S |
|  | ATCCCTGCTGGTGGT | AS |
| *CsacPBP2* | TGCGGACTTCTACAACTT | S |
|  | TGCCATCAGGGTCTAAC | AS |
| *CsacPBP3* | CGCTGATTCGGACAC | S |
|  | TCACCTCTACACTGGGAT | AS |
| *CsacOrco* | AACCAGTCAAACAGCCATCC | S |
|  | TACCCAACTAACAACAGACATCA | AS |
| *CsacOR1* | CTGCCTATTGCTCTTGGA | S |
|  | ATACTTTGTCAGGGAGATT | AS |
| *CsacOR2* | CATGGTGGTGAAGAAACG | S |
|  | TAATCCTCCGTGAAATGC | AS |
| *CsacOR3* | CGAGTTCATCGCCGAGTC | S |
|  | CCCGTAGCACCGCAATAT | AS |
| *CsacOR4* | CCCATTCTGGACTCTTCG | S |
|  | AGTTCCCACATTGTTTCG | AS |
| *CsacOR5* | CACTACCAGCATATCACAACT | S |
|  | AACTGAAGCTCCATAAGAAA | AS |
| *CsacOR6* | TGGTGCGGAACTCGTC | S |
|  | CTCGCCGTCCAAGAAG | AS |
| *CsacOR7* | TGCCTGCTTATGTTTGTTG | S |
|  | TACGGTTCTGTTCTATGATGTCT | AS |
| *CsacOR8* | AAATGGGAAGACGAGGCT | S |
|  | CACCGAAACAAGTTGGAAA | AS |
| *CsacOR9* | TGCCTGTTGTTCTGATGG | S |
|  | GGTATGGGCGGATAGTTG | AS |
| *CsacOR10* | TCAGGGAGACCTCCAGATAC | S |
|  | GTTGAGTGACGCAGAATACG | AS |
| *CsacCSP1* | TCGGAAGTCTTTAACCTACA | S |
|  | CACAATCTGCTGCCAATA | AS |
| *CsacCSP2* | AACACTGCCTGATGCTTT | S |
|  | TCCTTGTATCTTTGCTGGT | AS |
| *CsacCSP3* | GCTGTTATGGCTGTGGTTC | S |
|  | ATTTGCTGCATTCGGTCT | AS |
| *CsacCSP4* | GTGGCGTCAGTGGTTG | S |
|  | GTCGCACGGGTTCTTT | AS |
| *CsacCSP5* | CACCGAACACCCAAAGAC | S |
|  | CGTAAAGAATAACGGGAAGAC | AS |
| *CsacCSP6* | CTTCGCTATTTATCAACGC | S |
|  | CACATTTCGCTTCATCAC | AS |
| *CsacCSP7* | TCGGGAGAAGACTGAGAACA | S |
|  | TGGATGAAGGCGAGGGT | AS |
| *CsacCSP8* | TTGGCTCCGTTTGTTCT | S |
|  | ATCTGTCGCATTCTCCTG | AS |
| *CsacCSP9* | AAAGCAAATAGGGAAACAG | S |
|  | GTAATCCTGGAACGCATC | AS |
| *CsacCSP10* | CCGCCCACGATCAAAT | S |
|  | CGCAAGCCTGTTCAGTT | AS |
| *CsacCSP11* | AACTGCCTACTGGAGC | S |
|  | TCTTCGGGTCTGTGAT | AS |
| *CsacCSP12* | GACTGTTGGTTCCGTATGT | S |
|  | GCACCCTCCTTCTGTTT | AS |
| *CsacCSP13* | TTTGGACCGTTGGAT | S |
|  | AGTAACCTTGCCGTGA | AS |
| *CsacCSP14* | GTTGCCTCGTCGTTT | S |
|  | TCTCCACCGCTTCC | AS |
| *CsacCSP15* | TTTATTGTGCCTCGTCG | S |
|  | TAGCCTGCGGTTCTGC | AS |
| *CsacIR1* | CTCCGCCAGGAACAACC | S |
|  | CACGACGAACAGCCAGTAG | AS |
| *CsacIR2* | GCTTACGAGGATGGTGTC | S |
|  | GGCGGCGTTTAGGA | AS |
| *CsacIR3* | GAAGTTTGCCGAGGACA | S |
|  | GCGTTGATGGCTAAGAGTA | AS |
| *CsacIR4* | CAATGATTACTTGCCCTTAG | S |
|  | CCTGTCCGTTTCCTCC | AS |
| *CsacIR5* | TCCTGATGTGGGTGTTC | S |
|  | GGTTCTTTGGTGCCTC | AS |
| *CsacIR6* | CAGGAGACCACCTACAAACG | S |
|  | CACCTGGAAGGCGAAGAG | AS |
| *CsacIR7* | CCACCACCTTCCATCAA | S |
|  | TCTCAGTTCGTCCTCTATTTT | AS |
| *CsacIR8* | TAGCCCAGTCATCGTCG | S |
|  | GCTCGGATTCCTTCACC | AS |
| *CsacIR9* | CTACGAACCCTGACCACAT | S |
|  | GGGAAACCGATAAAGCATA | AS |
| *CsacIR10* | GACGCATTCTTGCCTGTA | S |
|  | AGCACCGTGGCTTTGG | AS |
| *CsacIR11* | AAGACATCGCCATACTTAGAG | S |
|  | CCCTTTGTTTGAGCACC | AS |
| *CsacIR12* | AAAAGATGTGCCCTCCA | S |
|  | TGACGCTGATTACTTGACC | AS |
| *CsacIR13* | CAAGTATTGCCACTATCACA | S |
|  | TTATCCACCTATCGCCTA | AS |
| *CsacSNMP1* | GGGAACGGATGGGACT | S |
|  | ACATAGCGGTTCGTCTTG | AS |
| *CsacSNMP2* | CTCTGGCTGCGTTACTGATT | S |
|  | TGATATGTTCGGCGTTGG | AS |

Note: S: sense primer; AS: antisense primer.
